# Risk factors for loss to follow‐up among at‐risk HIV negative men who have sex with men participating in a research cohort with access to pre‐exposure prophylaxis in coastal Kenya

**DOI:** 10.1002/jia2.25593

**Published:** 2020-10-01

**Authors:** Elizabeth W Wahome, Susan M Graham, Alexander N Thiong’o, Khamisi Mohamed, Tony Oduor, Evans Gichuru, John Mwambi, Elise M van der Elst, Eduard J Sanders

**Affiliations:** ^1^ KEMRI/Wellcome Trust Research Programme Centre for Geographic Medicine Research–Coast Kilifi Kenya; ^2^ Departments of Medicine, Epidemiology and Global Health University of Washington Seattle WA USA; ^3^ Department of Global Health University of Amsterdam Amsterdam the Netherlands; ^4^ Nuffield Department of Medicine University of Oxford Headington United Kingdom

**Keywords:** MSM, attrition, pre‐exposure prophylaxis, Kenya, LGBTQ, sub‐Saharan Africa, HIV

## Abstract

**Introduction:**

Retention in preventive care among at‐risk men who have sex with men (MSM) is critical for successful prevention of HIV acquisition in Africa. We assessed loss to follow‐up (LTFU) rates and factors associated with LTFU in an HIV vaccine feasibility cohort study following MSM with access to pre‐exposure prophylaxis (PrEP) in coastal Kenya.

**Methods:**

Between June 2017 and June 2019, MSM cohort participants attending a research clinic 20 km north of Mombasa were offered daily PrEP and followed monthly for risk assessment, risk reduction counselling and HIV testing. Participants were defined as LTFU if they were late by >90 days for their scheduled appointment. Participants who acquired HIV were censored at diagnosis. Cox proportional hazards models were used to estimate adjusted Hazard Ratio (aHR) of risk factors for LTFU.

**Results and discussion:**

A total of 179 participants with a median age of 25.0 years (interquartile range [IQR]: 23.0 to 30.0) contributed a median follow‐up time of 21.2 months (IQR: 6.5 to 22.1). Of these, 143 (79.9%) participants started PrEP and 76 (42.5%) MSM were LTFU, for an incidence rate of 33.7 (95% confidence interval [CI], 26.9 to 42.2) per 100 person‐years. Disordered alcohol use (aHR: 2.3, 95% CI, 1.5 to 3.7), residence outside the immediate clinic catchment area (aHR: 2.5, 95% CI, 1.3 to 4.6 for Mombasa Island; aHR: 1.8, 95% CI, 1.0 to 3.3 for south coast), tertiary education level or higher (aHR: 2.3, 95% CI, 1.1 to 4.8) and less lead‐in time in the cohort prior to 19 June 2017 (aHR: 3.1, 95% CI, 1.8 to 5.6 for zero to three months; aHR: 2.4, 95% CI, 1.2 to 4.7 for four to six months) were independent predictors of LTFU. PrEP use did not differ by LTFU status (HR: 1.0, 95% CI, 0.6 to 1.5). Psychosocial support for men reporting disordered alcohol use, strengthened engagement of recently enrolled participants and focusing recruitment on areas close to the research clinic may improve retention in HIV prevention studies involving MSM in coastal Kenya.

**Conclusions:**

About one in three participants became LTFU after one year of follow‐up, irrespective of PrEP use. Research preparedness involving MSM should be strengthened for HIV prevention intervention evaluations in coastal Kenya.

## 1 INTRODUCTION

Men who have sex with men (MSM) are among the populations at highest risk of HIV acquisition globally [1]. Incidence estimates of HIV in MSM in sub‐Saharan Africa (SSA) are 10 to 15 fold higher than in general populations in Africa: ranging from 5.1/100 person‐years (PY) (95% confidence interval [CI], 2.6 to 9.8) in Kenya to 15.4/100 PY (95% CI, 8.1 to 19.2) in Nigeria [2, 3, 4]. Pre‐exposure prophylaxis (PrEP) is effective for HIV prevention if adhered to [5, 6]. While PrEP provision to MSM is ongoing through research and programmes in some settings in SSA [7], retaining MSM in HIV prevention programmes is challenging in Kenya and other settings where same‐sex behaviour is criminalized [8, 9].

HIV vaccine feasibility cohorts offering PrEP and other prevention services to MSM provide helpful information on HIV incidence and retention. Among HIV‐negative MSM followed in such cohorts in Nairobi and coastal Kenya before PrEP rollout, high rates of loss to follow‐up (LTFU) have been documented [10, 11], with LTFU estimates as high as 42.2 [95% CI, 29.5 to 60.4] per 100 PY among men who have sex with men only [11]. With PrEP added as a biomedical prevention option offered at our clinic in 2017, we hypothesized that retention among MSM would be better than previously recorded in the same cohort. For this study, our objective was to estimate LTFU rates and assess risk factors of LTFU among at‐risk MSM participating in a cohort on the Kenyan coast after PrEP became available in June 2017.

## 2 METHODS

### 2.1 Study setting and population

Since July 2005, at‐risk individuals have been recruited for an open HIV vaccine feasibility cohort at the Kenya Medical Research Institute (KEMRI) clinic in Mtwapa, coastal Kenya. Located 20 km north of Mombasa, Mtwapa is known for its busy night life [12]. Participants were identified for recruitment by 10 to 15 trained peer mobilizers who approached individuals through personal networks and at venues where sex workers meet clients. While any man aged 18 to 49 years who reported anal sex in the three months before screening was eligible [11], peer mobilizers were encouraged to mobilize participants at elevated risk for HIV acquisition, including younger men (18 to 24 years of age) and those who reported receptive anal intercourse (RAI) or sex with men exclusively [11, 13]. This study includes all follow‐up visits between 19 June 2017 (when PrEP became freely available to participants) and 30 June 2019.

### 2.2 Cohort procedures

Procedures have been described elsewhere [11, 13, 14]. Briefly, all monthly visits included a face‐to‐face interview to assess risk behaviour, HIV counselling and testing using rapid antibody tests, medical history and physical examination. All participants were provided syndromic treatment for symptoms suggestive of sexually transmitted infections (STI), received care for minor illnesses as indicated and were vaccinated against hepatitis B.

Beginning in 2016, participants completed a yearly assessment for depressive symptoms (Patient Health Questionnaire 9 [PHQ‐9]), alcohol use (Alcohol Use Disorder Identification Test [AUDIT]), use of substances other than alcohol and tobacco (Drug Abuse Screening Test 10 [DAST‐10]), sexual stigma (abridged China MSM Stigma Scale) and recent trauma via audio computer‐assisted self‐interview (ACASI) in English or Swahili [15]. Following self‐assessment, participants debriefed with a counsellor. Participants who reported moderate or severe depressive symptoms, hazardous or harmful drinking, or moderate to severe abuse of other substances were engaged for supportive counselling at the KEMRI clinic or referred to local services.

Starting on 19 June 2017, free daily PrEP was provided according to Kenyan Ministry of Health (MoH) guidelines to participants eligible by MoH criteria or a cohort‐derived HIV risk score calculated at each visit [11, 13]. Participants eligible for PrEP and interested in taking it were provided with a 30‐day PrEP supply. During monthly visits, PrEP adherence and adverse effects, HIV status and syndromic STIs were assessed and refills provided. Participants who tested HIV‐positive discontinued PrEP and were engaged for HIV care and treatment. Those not taking PrEP were re‐assessed at each visit for eligibility and offered PrEP when found eligible [16].

### 2.3 Retention and tracing activities

At each visit, locator and contact information were verified and updated. Participants received 400 Kenyan shillings (KSh, ≈$3.87) for transportation and time at each monthly visit, per local research guidelines. An additional 100 KSh (≈$0.98) was provided when they reported on the scheduled appointment date. Participants were reminded of scheduled visits one week in advance and contacted by telephone within one day after a missed appointment. Those who could not be contacted by phone were physically traced, with up to three attempts made within a 14‐day window after the missed appointment.

### 2.4 Measures

#### 2.4.1 Loss to follow‐up

Participants were defined as LTFU if they were late by >90 days for their scheduled appointment date. Participants (N = 10) who re‐engaged before the censoring date after they were late by >90 days were defined as LTFU.

#### 2.4.2 Predictors of loss to follow‐up

##### Sociodemographic characteristics

**Figure 1:**
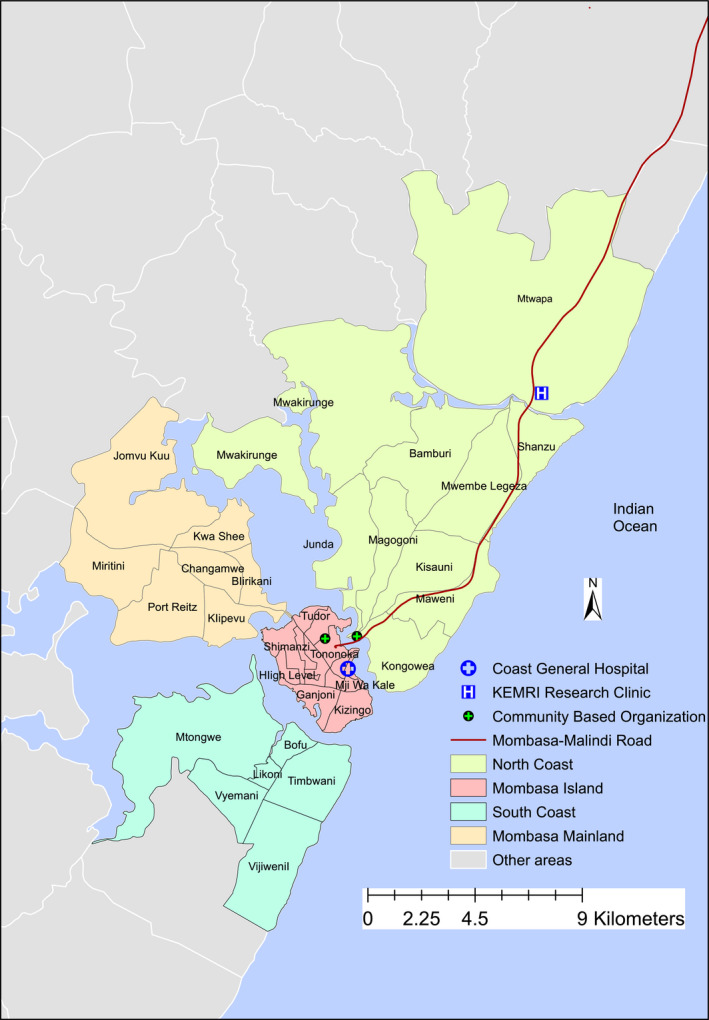
Map of study area in coastal Kenya, 2017 to 2019.

The following characteristics reported at enrolment were evaluated as potential risk factors: marital status, education, religion, employment status, earnings per month and years lived within the region. The study region was divided into four areas; north coast (closest to KEMRI clinic), Mombasa island (≈20 km distant), south coast (≈24 km distant requiring a ferry crossing) and Mombasa mainland and other more remote areas (≥26 km distant, Figure 1).

##### Time‐varying risk and mental health characteristics

The following time‐varying characteristics were evaluated as potential risk factors: age (18 to 24 vs. 25 + years), sex of sex partners in past three months (men and women or men only), anal sex role in past three months (receptive, versatile, insertive, no anal sex), condom use for anal sex in past three months (yes/no), number of sex partners in past week (none, one, ≥2), sexual behaviour in past week (no sexual activity, 100% condom use, <100% condom use for reported sex acts), receiving payment for sex in past three months (yes/no), paying for sex in past three months (yes/no), group sex in past three months (yes/no), moderate to severe depressive symptoms in past two weeks (PHQ‐9 score 10 to 27), disordered alcohol use in past year (AUDIT score ≥ 8), problematic substance use in past year (DAST‐6 score ≥ 1), sexual stigma score (score 0 to 33), recent trauma (score ≥ 1) in past one year, travel in past three months (yes/no) and follow‐up time in the cohort as of the June 2017 baseline (zero to three months, four to six months, >6 months). PrEP use was defined as receiving a PrEP refill at the previous visit and reporting continuation at the current visit (yes/no).

### 2.5 Statistical analysis

Descriptive statistics were used to summarize demographic, behavioural and mental health characteristics of participants at first visit after June 2017 in the study. For mental health data collected annually, the last observations were carried forward to monthly visits. Data for each participant were censored at their last monthly visit before the censoring date (30 June 2019) or LTFU. Participants (N = 10) who re‐engaged after being LTFU were censored at the last visit before being LTFU. Participants who acquired HIV infection during follow‐up were censored at the HIV diagnosis visit. Participants who attended only one visit (N = 9) were included in the analysis and assigned a follow‐up time of one day. Individual follow‐up time was calculated from 19 June 2017 until the last visit before 30 June 2019. Attrition rates were calculated as the number of LTFU cases divided by total PY of follow‐up and expressed as incidence per 100 PY. Cox proportional hazards models were used to assess risk factors for LTFU. Variables significant in bivariable analysis at *p* < 0.1 and age a priori were included in the initial multivariable model of potential predictors of LTFU. To reduce the number of predictors, only variables with *p* < 0.1 were retained in the final multivariable model. Tests of collinearity were run on the model and predictors with a variance‐covariance correlation of ≥0.5 were considered collinear and not included in the final multivariable model. *p* values were two‐sided, and significance was set at *p* < 0.05. Data were cleaned, recoded and analysed using Stata 15.0 (StataCorp LLC, College Station, TX, USA).

### 2.6 Ethical considerations

The KEMRI Ethics Review Committee approved the study. All participants provided written informed consent.

## 3 RESULTS AND DISCUSSION

### 3.1 Participants characteristics

Out of 179 participants followed during the study period, 177 (98.9%) were eligible for PrEP and 143 (79.9%) started it. At the first visit after PrEP became available, the median age was 25.0 years, interquartile range [IQR]: (23.0 to 30.0), more than half (52.0%) had a secondary education or higher. More than three‐quarters (76.0%) had lived in the study area for >1 year, nearly half (49.2%) resided in north coast (closest to research clinic), half (50.3%) qualified for the incentive for presenting on the scheduled appointment date and nearly two‐thirds (62.0%) had contributed >6 months of cohort follow‐up before PrEP became available (Table 1).

**Table 1:** Characteristics of 179 HIV negative Kenyan MSM at first visit following PrEP availability, June 2017–June 2019

Characteristics		All (n = 179)	LTFU (n = 76)	Not LTFU (n = 103)
		n (%)	n (%)	n (%)
Age group (years)				
18 to 24		72 (40.2)	34 (44.7)	38 (36.9)
25+		107 (59.8)	42 (55.3)	65 (63.1)
Education				
Primary/none		70 (39.1)	24 (31.6)	46 (44.7)
Secondary		93 (52.0)	43 (56.6)	50 (48.5)
Higher/tertiary		16 (8.9)	9 (11.8)	7 (6.8)
Marital status				
Never married		158 (88.3)	68 (89.5)	90 (87.4)
Ever married		21 (11.7)	8 (10.5)	13 (12.6)
Religion				
Christian		92 (51.4)	39 (51.3)	53 (51.5)
Muslim		45 (25.1)	18 (23.7)	27 (26.2)
Other/none		42 (23.5)	19 (25.0)	23 (22.3)
Employment				
None		30 (16.8)	12 (15.8)	18 (17.5)
Self		120 (67.0)	51 (67.1)	69 (67.0)
Formal		29 (16.2)	13 (17.1)	16 (15.5)
Earnings per month (100 KSh≈$0.98)				
≥10,000 (≈$98.0)		44 (24.6)	23 (30.3)	21 (20.4)
5,000 to 9,000 (≈$49.0‐≈$88.2)		91 (50.8)	41 (53.9)	50 (48.5)
<5,000 (≈$49.0)		44 (24.6)	12 (15.8)	32 (31.1)
Years lived in area				
≤1		43 (24.0)	22 (28.9)	21 (20.4)
>1		136 (76.0)	54 (71.1)	82 (79.6)
Area of residence				
North coast		88 (49.2)	27 (35.5)	61 (59.2)
Mombasa island		21 (11.7)	15 (19.7)	6 (5.8)
South coast		46 (25.7)	26 (34.2)	20 (19.4)
Mombasa mainland/other areas		24 (13.4)	8 (10.5)	16 (15.5)
Gender identity^b^				
Male		144 (80.4)	59 (77.6)	85 (82.5)
Transgender woman/other		32 (17.9)	15 (19.7)	17 (16.5)
Sex of sex partners, past three months				
Men and women		77 (43.0)	24 (31.6)	53 (51.5)
Men only		102 (57.0)	52 (68.4)	50 (48.5)
Anal sex role, past three months				
Receptive		51 (28.5)	24 (31.6)	27 (26.2)
Versatile		83 (46.4)	39 (51.3)	44 (42.7)
Insertive		38 (21.2)	12 (15.8)	26 (25.2)
None		7 (3.9)	1 (1.3)	6 (5.8)
Condom use for anal sex, past three months^a^		73 (40.8)	30 (39.5)	43 (41.7)
Sex partners, past week				
None		71 (39.7)	27 (35.5)	44 (42.7)
One		39 (21.8)	15 (19.7)	24 (23.3)
≥Two		69 (38.5)	34 (44.7)	35 (34.0)
Sexual behaviour, past week				
No activity		71 (39.7)	27 (35.5)	44 (42.7)
100% condom use		72 (40.2)	34 (44.7)	38 (36.9)
<100% condom use		36 (20.1)	15 (19.7)	21 (20.4)
Paid for sex with cash, living expenses or goods, past three months		31 (17.3)	14 (18.4)	17 (16.5)
Received payment for sex with cash, living expenses or goods, past three months		114 (63.7)	56 (73.7)	58 (56.3)
Group sex, past three months		3 (1.7)	1 (1.3)	2 (1.9)
Depressive symptoms (PHQ‐9), past 2 weeks^b^				
Minimal to mild (0 to 9)		113 (63.1)	45 (59.2)	68 (66.0)
Moderate to severe (10 to 27)		64 (35.8)	30 (39.5)	34 (33.0)
Disordered alcohol use (AUDIT), past year^b^				
Low (0 to 7)		112 (62.6)	39 (51.3)	73 (70.9)
Hazardous (8 to 40)		65 (36.3)	36 (47.4)	29 (28.2)
Problematic substance use (DAST‐6), past year^b^				
Yes (≥1)		138 (77.1)	66 (86.8)	72 (69.9)
Sexual stigma score (0 to 33) [Median (IQR)]^b^		6 (3 to 13)	6 (3 to 14)	6 (2 to 11)
Recent trauma, past year^b^				
None		67 (37.4)	27 (35.5)	40 (38.8)
Any		108 (60.3)	46 (60.5)	62 (60.2)
Travelled out of county, past 3 months^b^		105 (58.7)	44 (57.9)	61 (59.2)
Follow‐up time in cohort as of June 2017				
0 to 3 months		40 (22.3)	28 (36.8)	12 (11.7)
4 to 6 months		28 (15.6)	17 (22.4)	11 (10.7)
>6 months		111 (62.0)	31 (40.8)	80 (77.7)
Reported on appointment date		90 (50.3)	38 (50.0)	52 (50.5)
Recent sexually transmitted infection^c^		1 (0.6)	0 (0.0)	1 (1.0)

AUDIT, Alcohol Use Disorder Identification; DAST‐6, Drug Abuse Screening Test 6; IQR, interquartile ranges; LFTU, loss to follow‐up; PHQ‐9, Patient Health Questionnaire 9.

^a^ Among 172 MSM reporting anal sex in the past three months.

^b^ Missing 3 values for gender identity, PHQ‐9, AUDIT, DAST to 6 and travelled out of county; missing 4 values for recent trauma; missing 9 values for sexual stigma.

^c^ Defined as detection of Gram‐negative, intracellular diplococci in urethral or rectal secretions or rectal secretions or a new syphilis diagnosis within six months.

### 3.2 Loss to follow‐up and risk factors

Overall, participants were followed for a median of 21.2 months (IQR: 6.5 to 22.1), contributing 225.3 PY. Participants made a total of 2,822 scheduled follow‐up visits, of which 1,404 (49.8%) occurred on the scheduled appointment date. Nine participants acquired HIV during follow‐up, of whom 7 (66.7%) had contributed >6 months of cohort follow‐up before PrEP became available (HIV incidence: 3.8 per 100 PY (95% CI: 2.0 to 7.3)).

Seventy‐six (42.5%) participants became LTFU, for a crude incidence of 33.7 (95% CI, 26.9 to 42.2) per 100 PY. While MSM who were LTFU contributed a median follow‐up time of 4.5 months (IQR: 1.6 to 13.8), those retained contributed 22.1 months (IQR: 21.2 to 22.8). Of the 76 LTFU participants, 33 (43.4%) could not be contacted through telephone calls or physical tracing, 31 (40.8%) relocated to other towns outside the study area and 9 (11.8%) withdrew from the study. Ten (13.2%) of 76 participants who were LTFU re‐engaged in follow‐up before study censoring. Of 143 participants who started PrEP, 18 reported PrEP side effects: four (7%) among those who were LTFU and 14 (16%) among those who were retained (*p* = 0.09).

In bivariable analysis, LTFU was associated at *p* < 0.1 with, age (18 to 29 years), education, earnings, years lived within the area, area of residence, having sex with men only, sexual role taking, AUDIT score ≥8 and prior follow‐up time in cohort. Participants who became LTFU had similar reported PrEP use as those retained (IRR 1.0, 95% CI, 0.6 to 1.5). Receipt of incentive among participants who reported on the appointment date did not impact retention (IRR 1.0, 95% CI, 0.7 to 1.6). In the final multivariable analysis, participants who were LTFU were more likely to have a tertiary education level or higher, reside further away from the research clinic, report disordered alcohol use (AUDIT ≥ 8) and have participated in the cohort for a shorter time before PrEP became available than participants who were retained (Table 2).

**Table 2:** Risk factors for loss to follow‐up among 179 Kenyan MSM, June 2017–June 2019

Characteristics		LTFU, n = 76	Bivariable analysis	*p* value	Multivariable analysis	*p* value
		n/100PY [rate]	HR (95% CI)		aHR (95% CI)	
Age group (years)^a^						
18 to 24		31/63.0 [49.2]	1.5 (0.9 to 2.4)	0.085	1.3 (0.8 to 2.2)	0.338
25+		45/162.4 [27.7]	Reference		Reference	
Education^a^						
Primary/none		24/95.1 [25.2]	Reference		Reference	
Secondary		43/114.1 [37.7]	1.4 (0.9 to 2.4)	0.156	1.3 (0.7 to 2.1)	0.391
Higher/tertiary		9/16.2 [55.5]	2.0 (0.9 to 4.4)	0.090	2.3 (1.1 to 4.8)	0.024
Marital status						
Never married		68/198.7 [34.7]	Reference			
Ever married		8/26.6 [30.1]	0.9 (0.4 to 1.8)	0.692	–	–
Religion						
Christian		39/116.7 [33.4]	Reference			
Muslim		18/56.7 [31.8]	1.0 (0.6 to 1.7)	0.879	–	–
Other/none		19/51.9 [36.6]	1.1 (0.6 to 1.9)	0.750	–	–
Employment						
None		12/39.8 [30.1]	Reference			
Self		51/149.4 [34.1]	1.1 (0.6 to 2.1)	0.700	–	–
Formal		13/36.1 [36.0]	1.2 (0.5 to 2.7)	0.646	–	–
Earnings per month (100 KSh≈$0.98)						
≥10,000 (≈$98.0)		23/51.6 [44.6]	2.2 (1.1 to 4.4)	0.028	–	–
5,000 to 9,000 (≈$49.0 to ≈$88.2)		41/109.6 [37.4]	1.9 (1.0 to 3.6)	0.057	–	–
<5,000 (≈$49.0)		12/64.1 [18.7]	Reference			
Years lived in area						
≤1		22/44.9 [48.9]	1.6 (1.0 to 2.6)	0.070	–	–
>1		54/180.4 [29.9]	Reference			
Area of residence^a^						
North coast		27/123.0 [22.0]	Reference		Reference	
Mombasa island		15/18.2 [82.4]	3.4 (1.8 to 6.4)	<0.001	2.5 (1.3 to 4.6)	0.004
South coast		26/50.6 [51.4]	2.2 (1.3 to 3.7)	0.003	1.8 (1.0 to 3.3)	0.042
Mombasa mainland/other areas		8/33.5 [23.9]	1.1 (0.5 to 2.4)	0.851	0.9 (0.4 to 2.0)	0.709
Gender identity						
Male		60/177.3 [33.8]	Reference			
Transgender woman/other		15/46.4 [32.3]	1.0 (0.6 to 1.8)	0.984	–	–
Sex partners, past week						
None		25/72.6 [34.5]	Reference			
One		18/60.2 [29.9]	0.9 (0.5 to 1.7)	0.799	–	–
≥Two		33/92.6 [35.6]	1.0 (0.6 to 1.8)	0.855	–	–
Sexual behaviour, past week						
No activity		25/73.1 [34.2]	Reference			
100% condom use		39/94.8 [41.1]	1.2 (0.7 to 2.0)	0.470	–	–
<100% condom use		12/57.4 [20.9]	0.7 (0.3 to 1.3)	0.250	–	–
Sex of sex partners, past three months						
Men and women		26/113.3 [22.9]	Reference			
Men only		50/112.0 [44.6]	1.8 (1.1 to 2.9)	0.015	–	–
Anal sex role, past three months						
Receptive		23/50.6 [45.4]	1.2 (0.7 to 2.0)	0.487	–	–
Versatile		37/101.5 [36.4]	Reference			
Insertive		11/58.9 [18.7]	0.5 (0.3 to 1.1)	0.075	–	–
None		5/14.2 [35.2]	1.0 (0.4 to 2.5)	0.962	–	–
Condom use for anal sex, past three months						
No		36/116.0 [31.0]	Reference			
Yes		35/94.6 [37.0]	1.1 (0.7 to 1.8)	0.630	–	–
Paid for sex with cash, living expenses or goods, past three months						
No		64/196.1 [32.6]	Reference			
Yes		12/29.2 [41.0]	1.1 (0.6 to 2.0)	0.785	–	–
Received payment for sex with cash, living expenses or goods, past three months						
No		30/108.2 [27.7]	Reference			
Yes		46/117.1 [39.3]	1.3 (0.8 to 2.0)	0.245	–	–
Group sex, past three months						
No		75/219.0 [34.3]	Reference			
Yes		1/6.3 [15.8]	0.5 (0.1 to 3.7)	0.524	–	–
Depressive symptoms (PHQ‐9), past two weeks						
Minimal to mild (0 to 9)		48/154.2 [31.1]	Reference			
Moderate to severe (10 to 27)		27/69.5 [38.9]	1.1 (0.7 to 1.8)	0.586	–	–
Disordered alcohol use (AUDIT), past year^a^						
Low (0 to 7)		35/150.1 [23.3]	Reference		Reference	
Hazardous (8 to 40)		40/73.6 [54.4]	2.2 (1.4 to 3.5)	<0.001	2.3 (1.5 to 3.7)	<0.001
Problematic substance use (DAST‐6), past year						
No (0)		14/62.1 [22.5]	Reference			
Yes (≥1)		61/161.6 [37.8]	1.5 (0.8 to 2.8)	0.161	–	–
Sexual stigma score (0 to 33)		–	1.0 (1.0 to 1.0)	0.489	–	–
Recent trauma, past year						
None		28/107.7 [26.0]	Reference			
Any		46/115.9 [39.7]	1.4 (0.9 to 2.2)	0.181	–	–
Travelled out of county, past three months						
No		46/135.5 [33.9]	Reference		–	–
Yes		29/88.2 [32.9]	1.1 (0.7 to 1.7)	0.818	–	–
Follow‐up time in cohort as of June 2017^a^						
0 to 3 months		28/35.9 [78.0]	3.7 (2.2 to 6.1)	<0.001	3.1 (1.8 to 5.6)	<0.001
4 to 6 months		17/26.7 [63.7]	3.0 (1.7 to 5.3)	<0.001	2.4 (1.2 to 4.7)	0.009
>6 months		31/162.7 [19.1]	Reference		Reference	
Reported PrEP use						
No		36/90.8 [39.6]	Reference			
Yes		40/134.5 [29.7]	1.0 (0.6 to 1.5)	0.855	–	–
Reported on appointment date						
No		42/125.2 [32.7]	Reference			
Yes		35/100.1 [35.0]	1.0 (0.7 to 1.6)	0.885	–	–
Recent sexually transmitted infection^b^						
No		76/222.9 [34.1]	–	–		
Yes		0/2.4 [0.0]	–	–		

aHR, adjusted hazard ratio; AUDIT, Alcohol Use Disorder Identification; CI, confidence intervals; DAST‐6, Drug Abuse Screening Test 6; HR, hazard ratio; LTFU, loss to follow‐up; PHQ‐9, Patient Health Questionnaire 9; PrEP, pre‐exposure prophylaxis; PY, person‐years.

^a^ Factors significant at *p* < 0.1 in initial multivariable model (data not shown) and age a priori were included in the multivariable model.

^b^ Defined as detection of Gram‐negative, intracellular diplococci in urethral or rectal secretions or rectal secretions or a new syphilis diagnosis within six months.

In this study, we document a higher LTFU rate than previously reported in the pre‐PrEP period (33.7 vs. 23.9 per 100 PY) [11]. While PrEP interest [2, 17, 18, 19], and uptake [16] among MSM in SSA is high, PrEP concentrations among 34 MSM and 8 TGW assessed six months following PrEP initiation was low [20], suggesting that MSM face challenges in sustained daily PrEP taking. Therefore, alternative strategies are needed to support daily PrEP taking and to retain participants in prevention services [21].

Consistent with other studies among MSM in Africa [22], we previously reported a high proportion of hazardous alcohol use (44% to 45%) among MSM in Kenya [15, 23]. In this study, men reporting disordered alcohol use (AUDIT score ≥ 8) were more than twice as likely to become LTFU than those with lower AUDIT scores. These findings support the need to screen participants for harmful substance use and to facilitate individual or group supportive counselling services at the research clinic or referral to alcohol and substance use harm reduction, treatment and support organizations [24].

Men who had joined the cohort recently were more likely to be LTFU. Similarly, in a study conducted among HIV‐negative MSM in Brazil, the rate of LTFU was greatest in the first year of follow‐up [25]. While men without an altruistic reason to join a longitudinal study may soon lose interest in participating after their immediate needs (e.g. STI treatment, HIV testing) are met, our study suggests that participants need stronger support or access to additional services during their first few months of participation in order to be retained.

Participants residing further away from the KEMRI clinic were more likely to be LTFU. Distance and time taken to travel to the clinic likely contributed to missed study visits and LTFU [26]. Of note, 42% of participants who became LTFU reported that they had relocated outside the study area. While transactional sex was not associated with LTFU in our study, it was a predictor of missed visits among 609 HIV‐negative gay, bisexual and other MSM followed in a cohort study in Kisumu, Kenya [27]. MSM and male sex workers are often mobile, which may impact LTFU [10, 12]. Providing information about LGBTQ‐friendly PrEP‐providing facilities through peers or eHealth support [28], may improve continuity of care when participants move or decide to change clinics.

Compared to primary or no education, the rate of LTFU was two times greater for participants with tertiary education or higher. Why educated participants in our study were more likely to be LTFU is not clear. It is possible that they were more likely to have a formal employment, and their work schedule may have restricted them from keeping scheduled clinic visits. Flexible visit schedules and access to prevention services outside formal working hours may improve retention for more educated participants [29].

In this study, having sex with men only was not associated with LTFU. Previously, we documented that the most vulnerable participants for HIV acquisition were also those who reported having sex with men only (HIV incidence rates: 35.2 per 100 PY) [11]. Same‐sex behaviour is criminalized in most of SSA [8, 9], and MSM, especially those with no female partners, face stigma and discrimination [30, 31, 32]. While we did not collect data on sexual orientation for this study, gay men and men who are more publicly visible such as sex workers likely face greater stigma and discrimination in Kenya, and may require additional support to remain in PrEP care. Facilitating linkage of MSM participants to local LGBTQ organizations for support services may improve retention of study participants, especially when those organizations are included in research planning and implementation [33].

To mitigate LTFU, we have adopted a peer mobilization model, using trained peer educators to act as a link between participants and the research clinic. PrEP educational sessions led by peers and study staff are provided to improve PrEP knowledge and discuss barriers. A collaboration with a local LGBTQ organization was strengthened to facilitate retention and promote continuity of services for participants who move or disengage from research follow‐up. Strengthening engagement with participants during the study and targeted mobilizations of participants who reside in areas closer to the research clinic may also improve retention.

Our study had several limitations. First, the face‐to‐face behavioural interview may have been subject to social desirability bias. However, the questionnaire on mental health and substance use was conducted via ACASI and resulted in identification of an important factor (i.e. disordered alcohol use) associated with LTFU. Second, the last observations for mental health variables collected at yearly time points were carried forward to subsequent monthly visits and may have introduced misclassification bias. Third, we analysed LTFU as a binary outcome and did not assess number or patterns of missed visits. Of note, all participants made monthly visits, and the LTFU rate might be lower if only quarterly visits were required. Fourth, assessments of sexual behaviour at monthly visits used a 3‐month recall period, which may have led to over‐reporting of some exposures. Lastly, we could not determine reasons for LTFU or ascertain reengagement in PrEP care and other services among participants who were LTFU.

## 4 CONCLUSIONS

Our study documented a substantial LTFU from research participation. We identified disordered alcohol use, distance to research clinic, education level and prior follow‐up time in the cohort as risk factors for LTFU. These factors suggest that additional interventions to strengthen research participation are needed, including screening and services for alcohol use, and that greater engagement and support may be needed by newly enrolled participants. Further research is needed to assess if peer outreach in collaboration with local LGBTQ organizations will improve research retention.

## COMPETING INTERESTS

No competing interests were disclosed.

## AUTHORS’ CONTRIBUTIONS

ES and SG designed the research study. AT, KM and JM collected the data. TO and EW managed data. EW analysed data and wrote the original draft of the manuscript. EW, SG, AT, KM, TO, JM, EG, JM, EMvdE and ES reviewed and edited the manuscript. All authors have read and approved the final manuscript.
